# Synthesis and Characterization of a Pla Scaffold with Pseudoboehmite and Graphene Oxide Nanofillers Added

**DOI:** 10.3390/nano15030167

**Published:** 2025-01-22

**Authors:** Rafael Vieira Maidana, Antônio Hortêncio Munhoz, Filipe Figueiredo Ramos, Alex Lopes de Oliveira, José César de Souza Almeida Neto, Victor Inácio de Oliveira, Bruno Luis Soares de Lima, Fábio Jesus Moreira de Almeida

**Affiliations:** Engineering School, Mackenzie Presbyterian University, 930 Consolação Street, Consolação, São Paulo 01302-907, SP, Brazil; rafael.maidana@mackenzista.com.br (R.V.M.); antonio.munhoz@mackenzie.br (A.H.M.J.); filipe.ramos@mackenzie.br (F.F.R.); alex.oliveira@mackenzie.br (A.L.d.O.); jose.almeida@mackenzie.br (J.C.d.S.A.N.); victor.inacio@mackenzie.br (V.I.d.O.); bruno.lima@mackenzie.br (B.L.S.d.L.)

**Keywords:** nanocomposites, polymeric composites, nanomaterials, PLA scaffold, graphene oxide, pseudoboehmite, skin grafts

## Abstract

In cases of severe injuries or burns, skin grafts (scaffolds) are often required as skin substitutes. In order not to harm the patient or the donor, biodegradable and biocompatible materials are used, which validates the search for heterografts such as poly (L-lactic acid)—PLA. However, natural polymers applied to the skin suffer great degradation in environments with large amounts of carbon and water or via binders with considerable resistivity, which implies little durability due to their low ductility. For the proposal, this work investigates PLA-based scaffolds modified with a mixture of pseudoboehmite (PB) and graphene oxide (GO), produced via the sol–gel route. The nanomaterials are incorporated into the polymer at different loadings, seeking to improve mechanical and thermal properties. Analyses via SEM, EDS, and XRD confirm the presence and distribution of these fillers. Tensile and flexural tests indicate that adding the filler can increase stress resistance, prevent deformations before failure, and increase toughness when compared to pure PLA.

## 1. Introduction

More than a million people suffer burns each year in Brazil [1]; however, less than 100,000 become injured at a level that is necessary for surgery and/or graft insertion. Skin grafting is a common surgical procedure used in various medical fields, particularly in wound healing and reconstructive surgery. Recent advancements in engineering regenerative skin grafts have highlighted the importance of biomaterials in achieving scarless wound healing [2]. The heterograft is a tissue or bone graft that is ideal for hosting biological components, ensuring their permanence at the site of the injury and assisting in its medicinal actions.

Biocompatible materials are materials that can interact with the living body, tissues, and cells without causing harmful effects [3]. Natural biopolymers like collagen, gelatin, and chitosan have been extensively utilized in commercial skin grafts due to their biocompatibility and effectiveness in promoting skin regeneration [4].

Among biodegradable polymers, thermoplastics are a group of materials that can degrade in environments containing carbon dioxide, water, and biomass, such as human tissue. These polymers can undergo degradation and exhibit low tenacity [5]. Polyolefins such as polyethylene (PE) and polypropylene (PP) are commonly used as thermoplastics due to their good mechanical and processing properties, low cost, non-toxicity, and recyclability, making them highly desirable for a wide range of applications [6].

Poly(lactic acid) (PLA) is a bio-based polymer widely used in various commercial applications [7]. PLA has attractive mechanical properties, renewability, biodegradability, and relatively low cost, making it a frontrunner in biopolymers [8]. Nanocomposite technology has been used to explore the use of PLA for various end-use applications. Ref. [9] focuses on the fabrication of bioactive nanocomposites using PLA and graphene oxide (GO) for potential applications in tissue engineering. Ref. [10] explores the mechanical and thermal properties of nanocomposites made from graphene oxide-graft-poly(l-lactide) and poly(l-lactide), shedding light on the potential applications of these materials. Ref. [11] discusses the significant improvement in the mechanical properties and heat distortion resistance of PLA when combined with functionalized reduced GO.

Ref. [12] discusses the preparation, properties, and applications of polymer/boehmite alumina (BA) nanocomposites. Ref. [12] details the synthesis techniques for BA, including solid-state decomposition, precipitation, and sol–gel processes. The primary objective of the incorporation of BA into polymers is to enhance the mechanical, thermal, and barrier properties of those polymers. In this context, ref. [13] investigates the enhancement of the mechanical, thermal, and fire properties of PLA composites by incorporating boehmite alumina.

Ref. [14] investigates the design and development of 3D printable boehmite alumina and thermally exfoliated reduced graphene oxide-based polymeric nanocomposites with high-dielectric-constant, mechanical, and thermomechanical performance. Ref. [15] investigated the synthesis, characterization, and properties of hybrid boehmite–graphene-oxide-filled epoxy composites. The study aimed to enhance the mechanical and thermal properties of epoxy resin by integrating graphene oxide and surface-modified boehmite nanorods.

In this work, heterograft precursor materials were obtained from compounds formed by biodegradable polymers structured with GO nanoparticles and pseudoboehmite (PB) particles. The main intention is to improve current scaffolds in terms of mechanical properties, preserving thermal properties so as not to affect degradation.

The main problems encountered are tissue acceptance by the patient, film breakage due to low ductility, and fragility in placement and extraction techniques among others. As the evolution of medicine is directly linked to the evolution of engineering, especially materials engineering, it is possible to design, develop, process, and characterize technological biomaterials that can serve as scaffolds as alternatives for some cases of injuries [16].

### Motivation

Related studies in Brazil employ animal skin for treatment [17], which can lead to scaffold rejection, contamination, compliance issues with the Brazilian Health Regulatory Agency (ANVISA), and ethical debates. To avoid these problems, a national solution that does not depend on animal-derived materials is necessary, prompting the use of composite materials and biomedical engineering syntheses. In previous work, the authors focused on synthesizing and comparing PLA/PB and PLA/GO. This study builds upon those findings by synthesizing PLA/GO/PB scaffolds.

## 2. Materials and Methods

Pseudoboehmite is a nanomaterial composed of aluminum, which has a structure similar to boehmite (γ-AlO(OH)). It is a material that is normally used in powder form, which has ceramic characteristics, and it is normally synthesized via the sol–gel process, a material synthesis process in which there is a transition from the sol system (dispersion of colloidal particles of sizes between 1 and 100 nm and that are stable in a fluid) to a gel system (system formed by the rigid structure of colloidal particles). This method allows for the control of physicochemical characteristics and thus can obtain pure pseudoboehmite [18].

Pseudoboehmite’s main characteristic is its highly porous surface, which affects its specific surface area [19]. This property means that this material is used in various applications, such as the controlled release of drugs in an organism [20]. Regarding toxicity, pseudoboehmite has shown low toxicity even in situations of high-concentration applications, therefore being characterized as a non-toxic agent [21,22].

GO is another nanomaterial of great interest, being obtained from the exfoliation of graphite oxide. After the chemical exfoliation and washing process, the stacking of the graphene planes of graphite oxides is reduced to sp2 hybrid carbon mono-layer structures, which may present hydroxyl, carbonyl, and epoxy functional groups on the surface and at the ends of the carbon sheet. These functional groups are responsible for modifying the properties of the materials when GO is used as a coating or structuring agent: for example, making the matrices present better conductivity without thermal properties being affected and improving the loss of heat properties. These functional groups also facilitate interactions with other materials, contributing to the production of polymer composites. However, they can interfere with the electrical properties of the final compound [23,24].

The PLA polymer is obtained from an acid of biological and, consequently, organic origin, coming from renewable resources. PLA can be obtained via fermentation from a series of microorganisms and is widely used in the synthesis of a series of polymers [25]. Lactic acid is a chiral molecule (contains asymmetric carbons) with two stereoisomers, L- and D-lactic acid [26]. PLA is the result of the polymerization of monomer lactic acids.

PLA bodies, with PB and GO added separately, have already been reported in the work of [27], where the following was concluded:

(a) In the PLA samples with PB added, there was an increase in heat capacity and density, which delays thermal degradation, accompanied by an increase in the modulus of elasticity and thus an increase in toughness, making it less rigid in relation to pure PLA.
(b) However, in PLA samples with GO added, in addition to the cost/benefit aspect, they exhibited more tenacity than PLA + PB samples and greater tensile and impact resistance than pure PLA and PLA + PB; however, they resist heat less than the PLA + PB composition.

### Synthesis of Nanomaterials and Production of PLA Compositions with GO+PB

GO was prepared by exfoliating graphite in a 98% concentrated sulfuric acid solution with the presence of sodium nitrate, and potassium permanganate was also dispersed to intercalate oxygen between the graphite planes. Based on [28], hydrogen peroxide was added to weaken the bonds and facilitate the unstacking of planes, forming graphite oxide. After chemical exfoliation and washing, the stacking of the graphene planes of graphite oxide was reduced to sp^2^ hybrid carbon monolayer structures [29], which may present hydroxyl, carbonyl, and epoxy functional groups on the surface and at the ends of the carbon sheet, which characterize GO.

The synthesis of pseudoboehmite occurred using the sol–gel method, incorporating a graphene oxide solution into the synthesis components. This procedure aims to better mix the nanomaterials, where the pseudoboehmite covers the graphene oxide, which can generate different behaviors in the mixture when used as a filler.

First, 562.5 g of aluminum nitrate was dissolved in 600 mL of GO solution with deionized water using a magnetic stirrer in order to promote homogeneity at the atomic level. The solution obtained was then dripped into 342 mL of ammonium hydroxide solution, generating a gel with the desired charge [30].

The pH of the solution must be kept alkaline at approximately nine during the process; however, due to the GO solution having an acidic character, the pH was reduced several times. Therefore, it was necessary to add ammonium hydroxide to correct the pH, with approximately an additional 300 mL. After dripping, a filtration process was carried out using a vacuum pump, and the gel was washed with deionized water until the filtered liquid reached a neutral pH (pH = 7). Approximately 25.3 g of the sample of the gel filler was separated and lyophilized during preparation for characterization assays.

The compositions were produced using polylactic acid (PLA) in pellets and the filler initially in gel, making 3 compositions: pure PLA, PLA + 3% filler by mass, and PLA+5% filler by mass. For each composition, an initial mass of approximately 1.5 kg of the polymer was used, with the 3% composition using 285.5g of the gel filler and the 5% composition using 474.9 g of the gel filler.

For filled compositions, the gel was initially manually mixed with the polymer in pellets, using spatulas and a tray, until homogeneous. After this, the pure PLA composition was plasticized in a heated rotary mixer at a temperature of 180 °C, generating a uniform mass for producing test specimens.

Then, the 3% composition was mixed in a mixer without success. The presence of water in the mixture, resulting from the gel washing process, generated steam accumulation during the process, preventing correct mixing. The material was relocated to a tray; then, both filled compositions were dried in an oven at 30 °C for approximately 24 h until the gel became a fine powder.

Once this was carried out, the process was tried again in the mixer, in which the 3% composition was successful, with the load spreading well, although it did not melt completely. The 5% composition, however, was still slightly wet, so the mixing process failed, with the filler accumulating in dry flakes rather than dispersing into the polymer.

These flakes were separated using tweezers and ground in a mortar until they became a fine powder, which was then mixed back into the polymer. The thermoplastic mixture was tried again, and it was successful this time, with a larger part of the polymer melted compared to the 3% composition.

## 3. Results and Discussion

**Figure 1:**
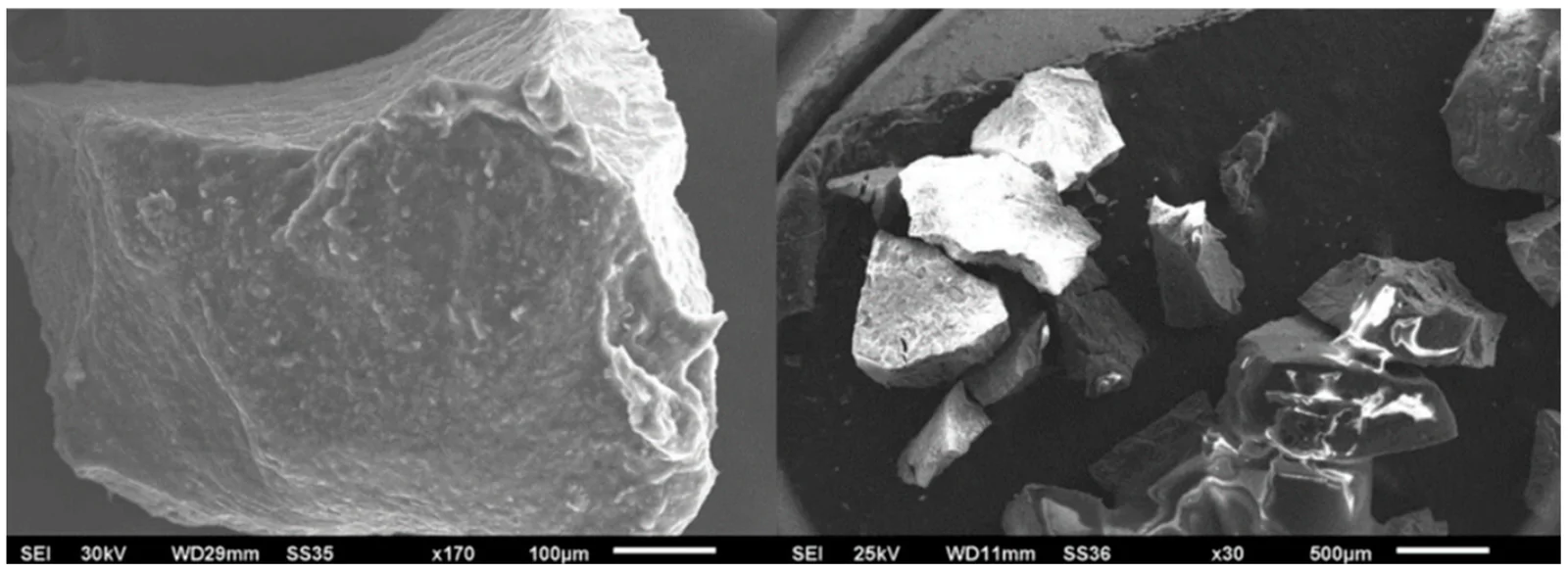
SEM image with 30× and 170× magnification.

The analysis of the specimens was generated via a SEM. The generated images had a magnification resolution of 30× and 170×, with a resolution of 500 µm and 100 µm, respectively, allowing the surface of the PB particles to be observed. However, higher magnifications had poor contrast, resulting in fuzzy images. The SEM images are shown in Figure 1.

**Figure 2:**
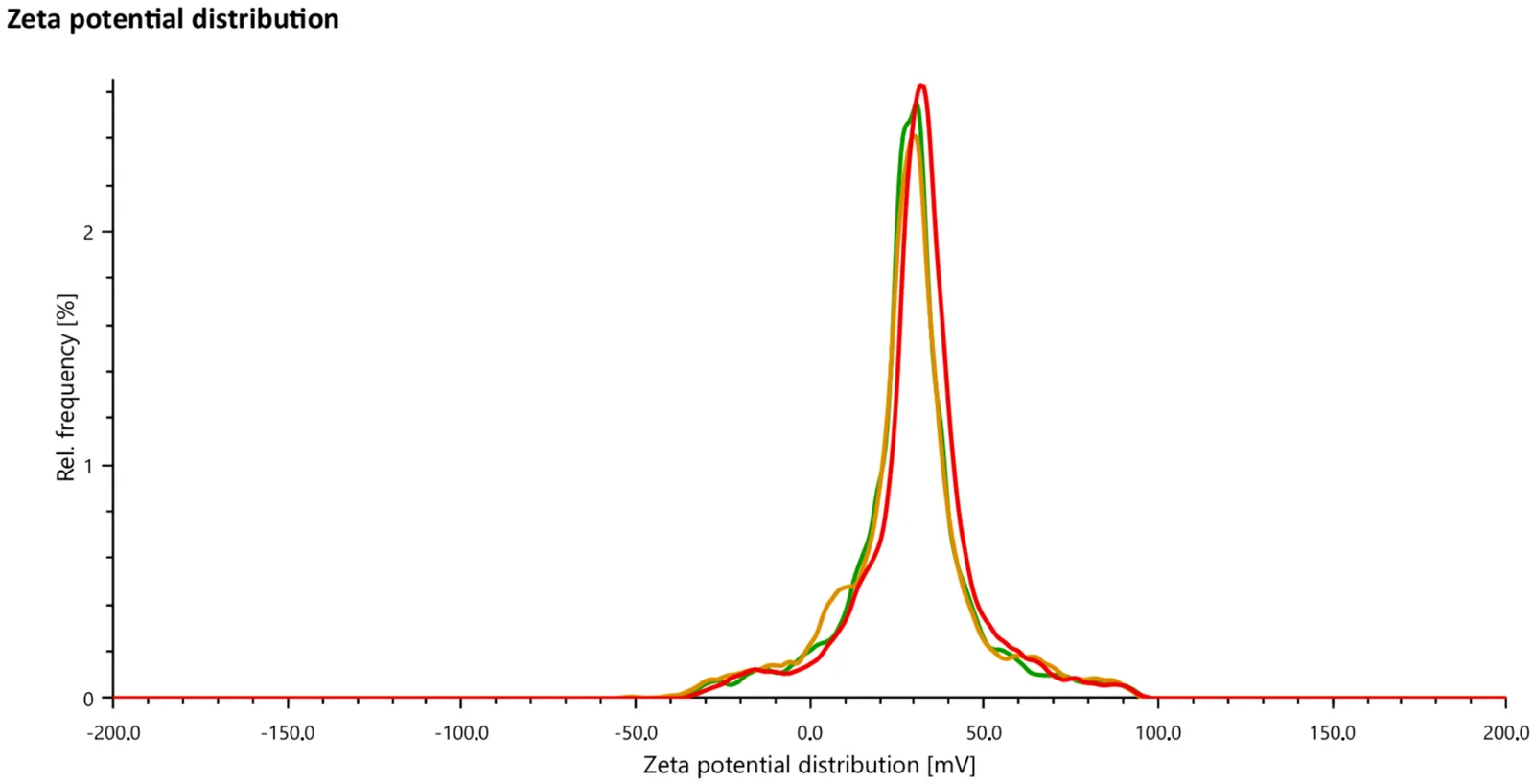
Zeta potential analysis results.

The zeta potential analysis showed a potential that is measured close to 30 mV; therefore, according to the literature, it presents the good stability of the particles, as mentioned in [31,32]. The results obtained in the zeta potential test are shown in Figure 2 and Table 1.

**Figure 3:**
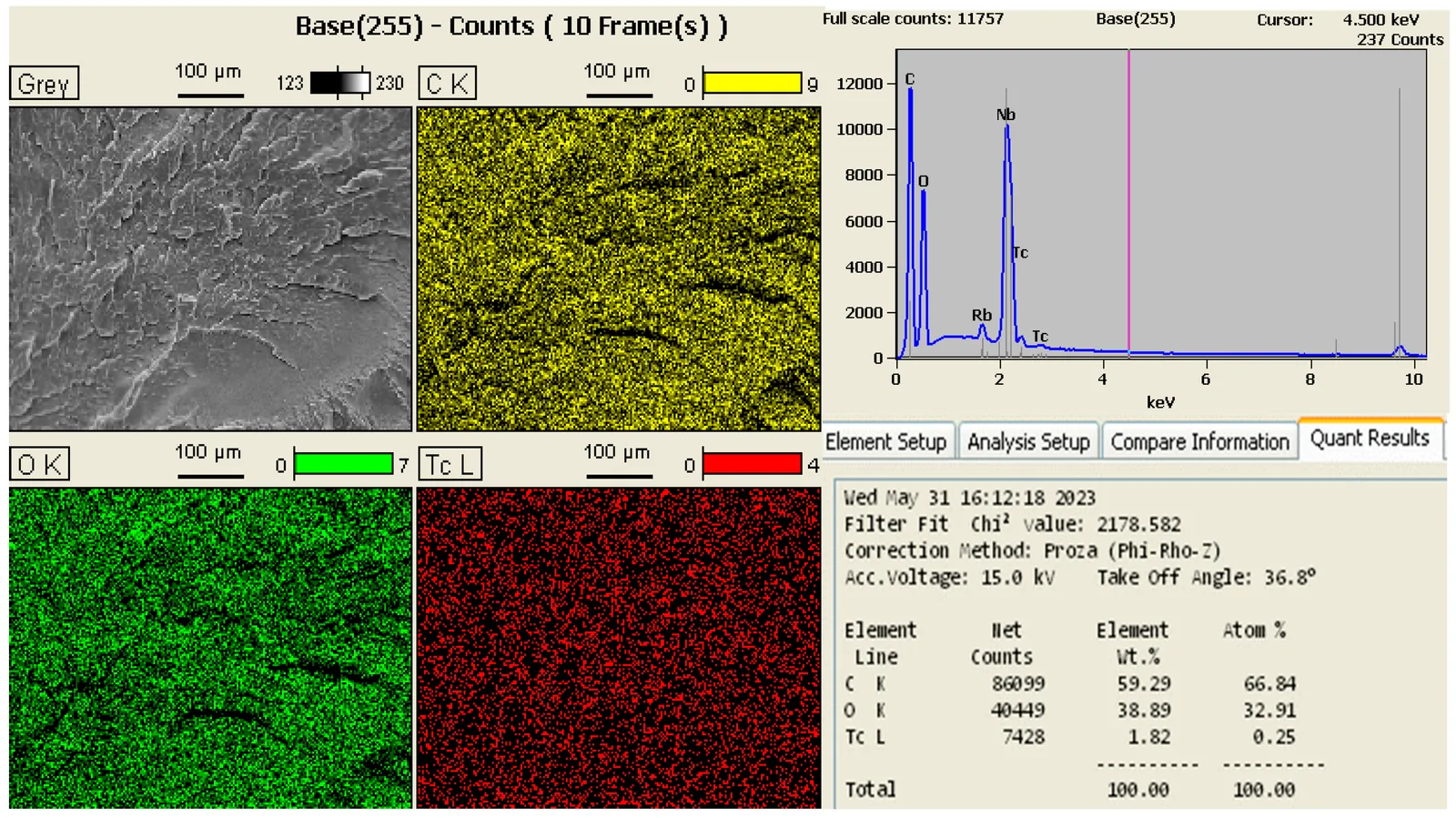
Results of the GO EDS analysis.

The Energy Dispersive Spectroscopy (EDS) analysis of graphene oxide (GO) showed that the analyzed material was predominantly composed of carbon and oxygen. The EDS software also generated small automatic indications assigned to rubidium (Rb) and technetium (Tc). However, these assignments were not independently confirmed. Both indications occurred in spectrally crowded regions susceptible to peak overlap, background effects, low-count statistical noise, and automatic misidentification. The non-conductive sample was coated with gold before SEM/EDS analysis, and the Au M-lines provide a known spectral contribution in the same low-energy region. In addition, technetium is not chemically expected in commercial GO, PLA, pseudoboehmite, or the reagents and preparation materials used in this study, and no plausible technetium source existed in the experimental process. The corresponding software-generated ‘Tc L’ map was weak, speckled, and showed no coherent spatial correlation with the sample morphology. Accordingly, neither the Rb nor the Tc indication should be interpreted as evidence that these elements were genuine constituents of the sample. They are therefore treated as unconfirmed automatic software assignments/artifacts, and the material is interpreted as being predominantly composed of carbon and oxygen, as presented in Figure 3.

**Figure 4:**
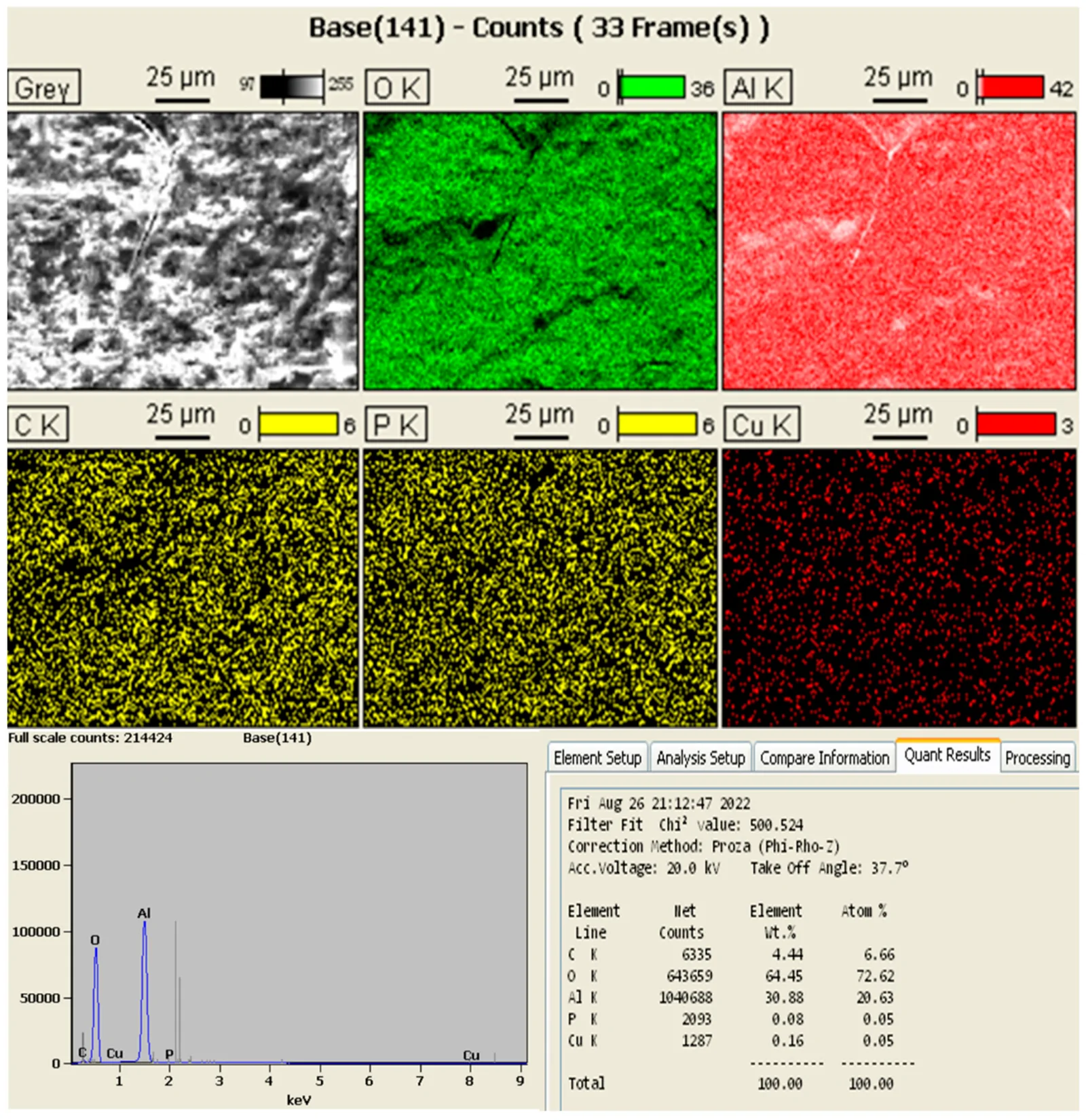
Results of the EDS analysis of the nanofiller.

**Figure 5:**
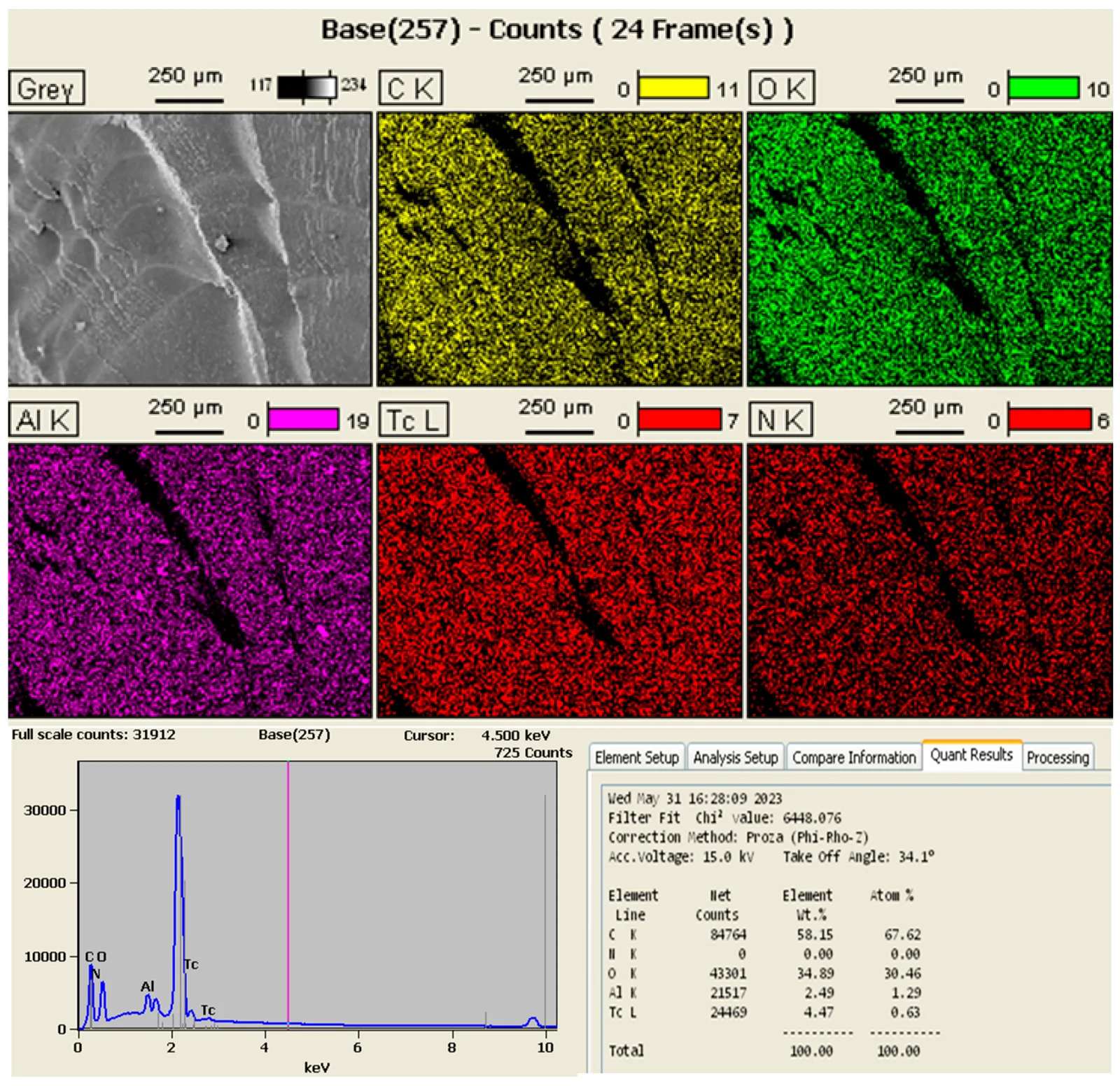
Results of the EDS analysis of the nanomaterial.

The EDS analysis of the nanofiller in turn demonstrated that the sample indeed contained the elements carbon, oxygen, and aluminum, indicating that the mixture of PB and GO was successful. The elements phosphorus (P) and copper (Cu) were also found in considerably lower quantities, probably being impurities that joined the material during some stage of its processing. The results of the EDS analysis are shown in Figure 4 and Figure 5.

The IZOD impact test resulted in average values of absorbed energy, energy per thickness, and energy per surface, with the data obtained shown in Table 2, Table 3 and Table 4.

Only one of the specimens did not present a complete fracture, this being one of the bodies in the 3% load composition with a hinge-type fracture.

For all these values, the composition of PLA + 5% (PB+GO) presented superior results than those of the pure polymer, while PLA + 3% (PB+GO) presented inferior results to the pure material, indicating that the material was weakened.

The hinge-type fracture, by allowing a localized region of plastic deformation, indicates that the material has a certain capacity for energy absorption and stress redistribution prior to complete failure. This behavior not only suggests greater impact resistance, but it is also tied to tensile and flexural properties, as the ability to withstand localized deformation is often linked to good interfacial adhesion among the composite constituents. Furthermore, the way the fillers (fibers or particles) are dispersed throughout the matrix can promote this fracture mechanism, preventing the immediate propagation of cracks and enabling the formation of a “hinge zone”. Consequently, both the microstructure (distribution, size, and shape of the fillers) and the quality of their interaction with the matrix directly influence the fracture mode, underscoring important correlations among impact, tensile, and flexural performance.

The tensile tests revealed that the addition of the load improved both the average stress supported and the total deformation in relation to the pure material. The 3% filler composition showed a small improvement in the average supported stress from 30.2 MPa to 33.5 MPa, as well as a slight increase in deformation from 1.2% to 1.6%. The composition with the 5% load showed a greater increase in supported stress from 30.2 MPa to 39.1 MPa, as well as greater deformation from 1.2% to 1.9%.

**Figure 6:**
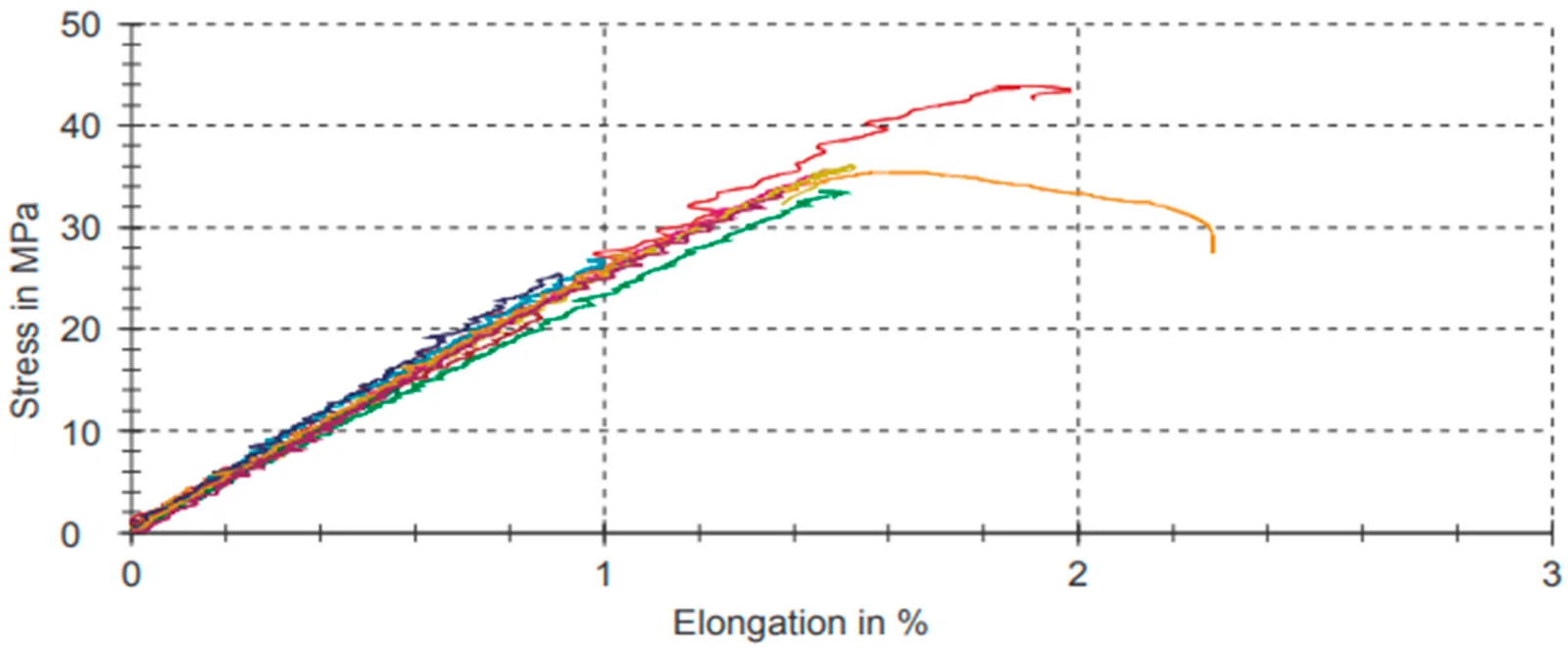
Strain–stress graphic for pure PLA.

Table 5 shows the data results of the strain–stress relative to pure PLA bodies. Figure 6 shows graphs referring to the analyses of pure PLA bodies represented in Table 5.

Table 6 shows the data results of the strain–stress test for compositions with the 3% filler of PB-GO.

**Figure 7:**
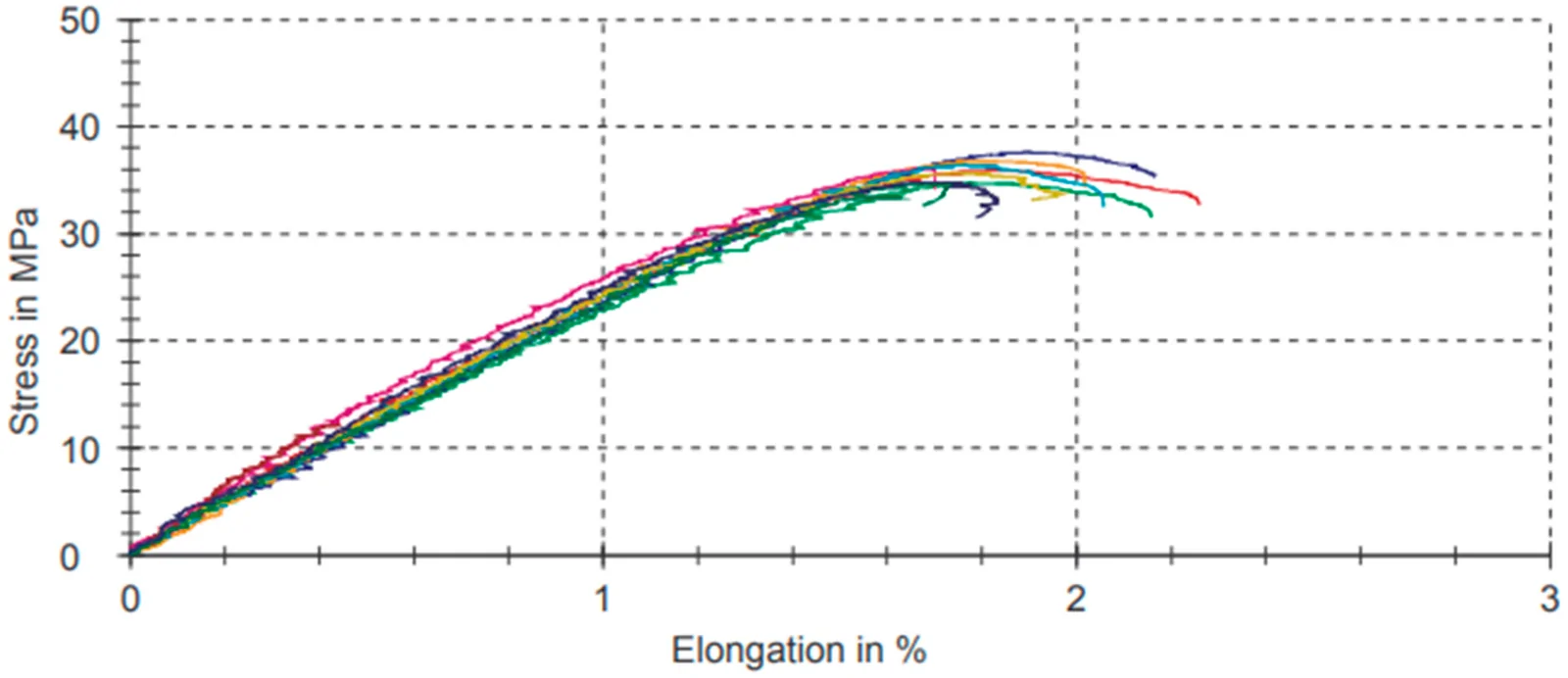
Strain–stress graphic for compositions with the 3% filler of PB-GO.

Figure 7 shows the graphs referring to the analyses for compositions with the 3% filler of PB-GO represented in Table 6.

Table 7 shows the data results of the strain–stress test for compositions with the 5% filler of PB-GO.

**Figure 8:**
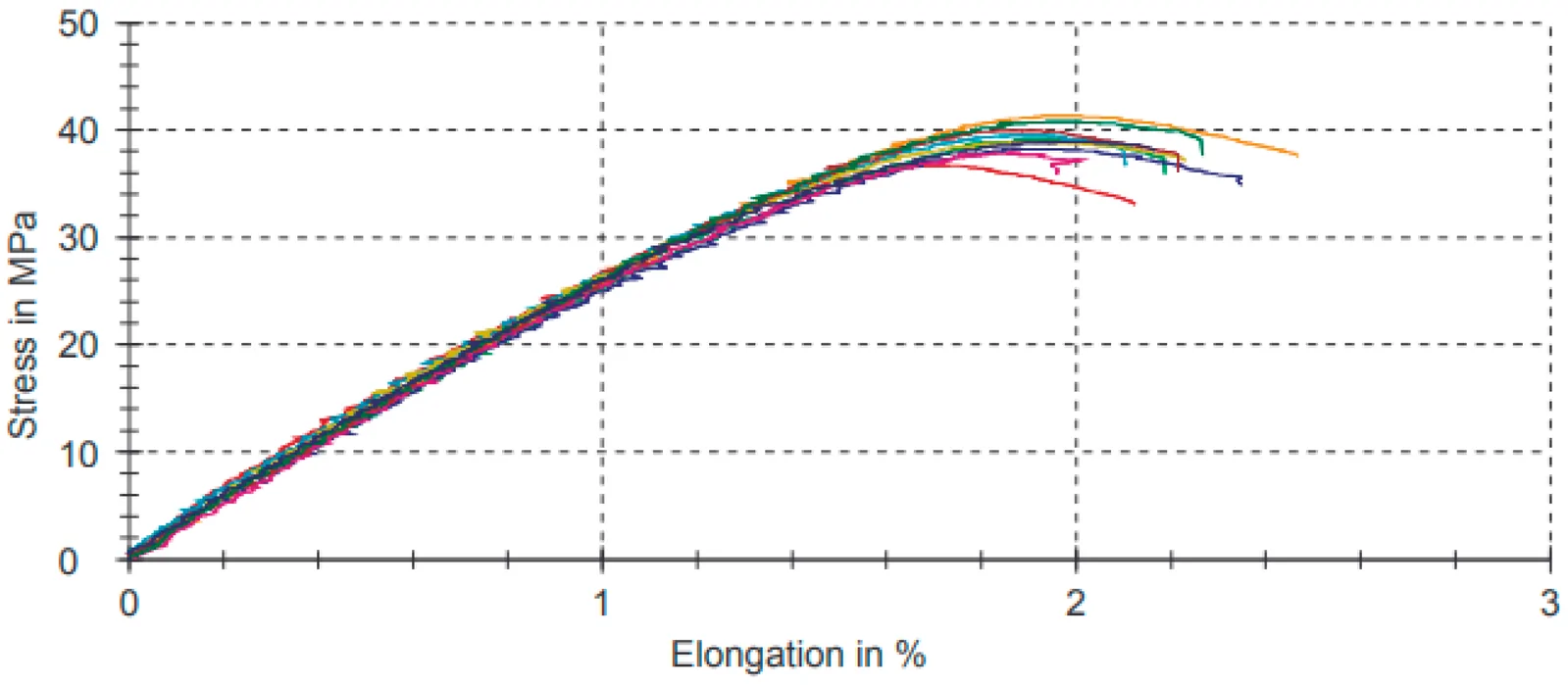
Strain–stress graphic for compositions with the 5% filler of PB-GO.

Figure 8 shows the graphs referring to the analyses for compositions with the 5% filler of PB-GO represented in Table 7.

**Figure 9:**
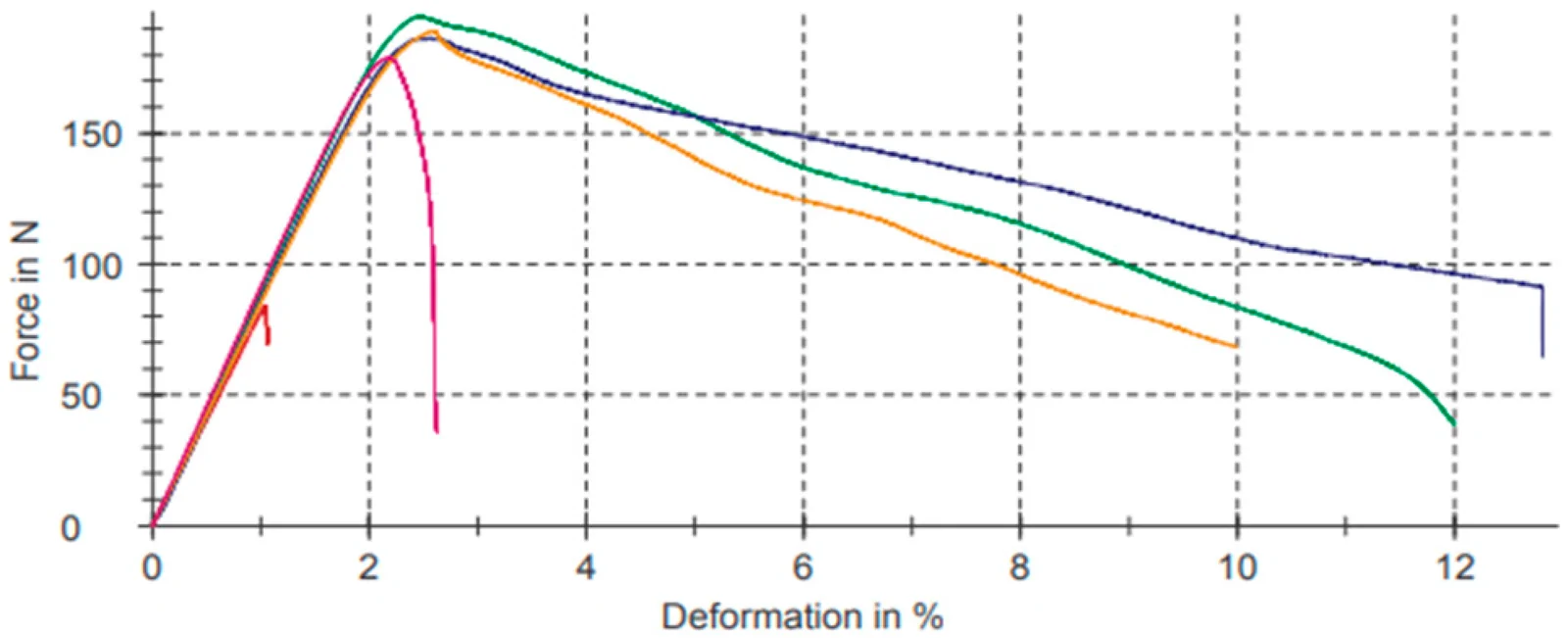
Bending test for composition with pure PLA.

**Figure 10:**
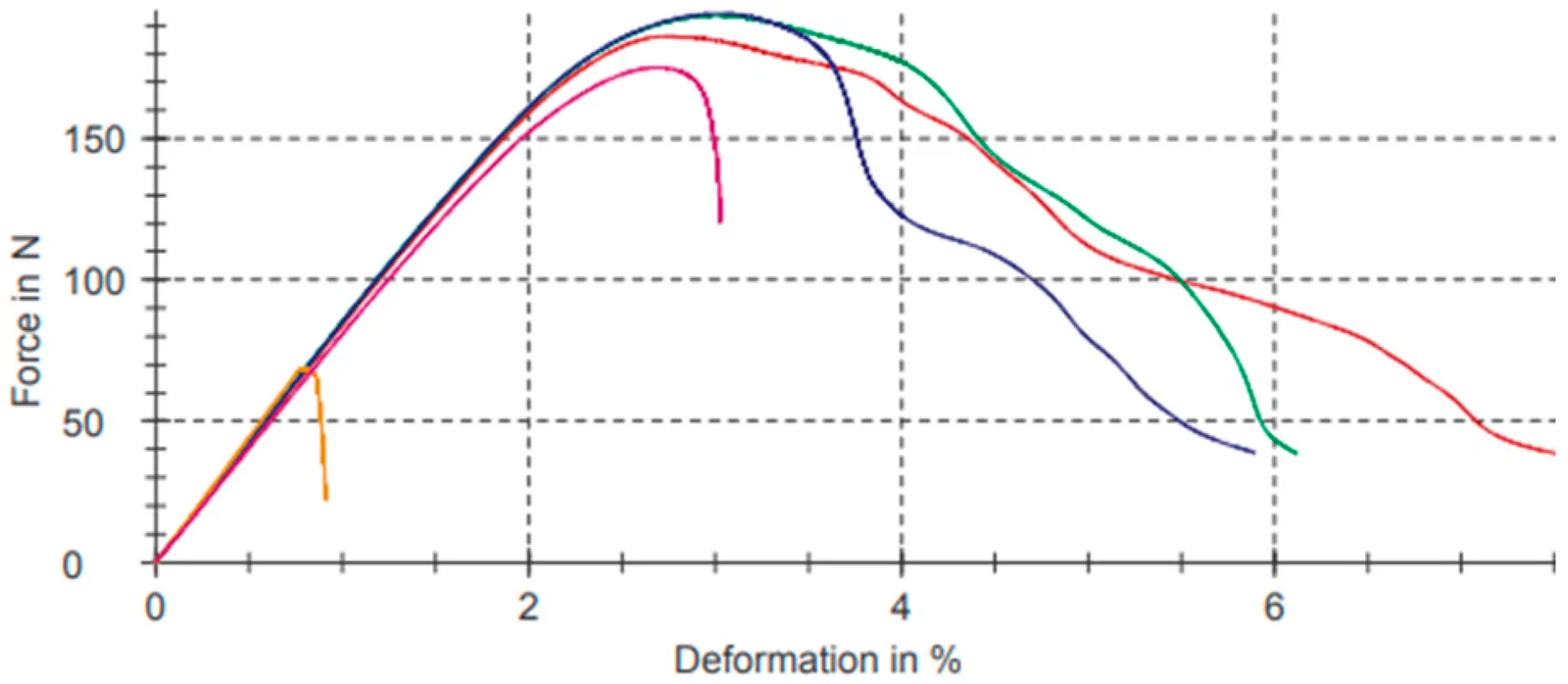
Bending test for composition with the 3% filler of PB-GO.

**Figure 11:**
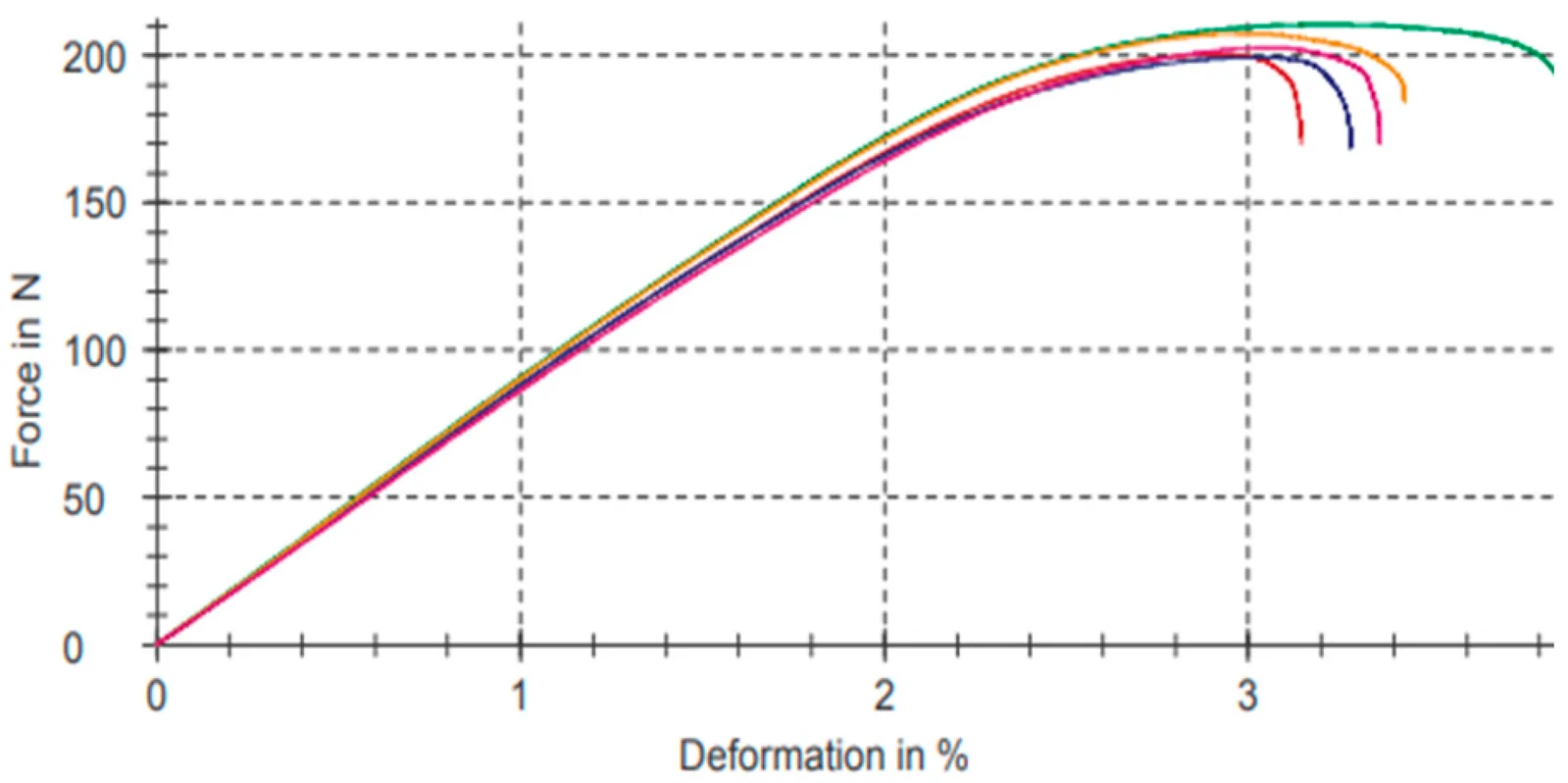
Bending test for the composition with the 5% filler of PB-GO.

The flexural tests demonstrated that the addition of the load resulted in a small drop in the average supported stress value from 50.6 Mpa to 49.7 Mpa and a slight increase in the deformation suffered before failure from 2.2% to 2.4% for the composition with the 3% filler. For the 5% load composition, there was an increase in both the average supported stress from 50.6 Mpa to 61.9 Mpa, and the deformation suffered from 2.2% to 3%. These results, combined with the results of the tensile and impact tests, indicate that the 5% composition performed considerably better than the 3% composition in improving the mechanical properties of PLA. The results of the bending tests are shown in Figure 9, Figure 10 and Figure 11. Table 8 shows data from bending tests for pure PLA.

Figure 9 shows the graphs referring to bending test results for the composition with pure PLA represented in Table 8.

Table 9 shows data from bending tests for compositions with the 3% filler of PB-GO.

**Table 9:** Bending test results for compositions with the 3% filler of PB-GO.

	$E_{r}$	$\sigma_{0.2}$	$\sigma_{fY}$	$\varepsilon_{fY}$	$\sigma_{fM}$	$\varepsilon_{fM}$	$\sigma_{fB}$	$\varepsilon_{fB}$	h	b	$A_{0}$
No.	MPa	MPa	MPa	%	MPa	%	MPa	%	mm	mm	${mm}^{2}$
1	2590.00	52.0	56.5	2.7	56.5	2.7	11.3	7.6	6.08	13.38	81.35
2	2630.00	52.6	58.9	3.0	58.9	3.0	11.8	6.1	6.07	13.38	81.22
3	2650.00	53.1	59.4	3.0	59.4	3.0	11.9	5.9	6.06	13.37	81.02
4	2640.00	17.6	59.6	3.0	20.2	0.8	6.44	0.9	6.15	13.45	82.72
5	2490.00	49.1	59.6	3.0	53.3	2.7	36.7	3.0	6.07	13.37	81.16

Figure 10 shows graphs referring to bending test results for the composition with the 3% filler of PB-GO represented in Table 9.

Table 10 shows data from bending tests for compositions with the 5% filler of PB-GO.

**Table 10:** Bending test results for compositions with the 5% filler of PB-GO.

	$E_{r}$	$\sigma_{0.2}$	$\sigma_{fY}$	$\varepsilon_{fY}$	$\sigma_{fM}$	$\varepsilon_{fM}$	$\sigma_{fB}$	$\varepsilon_{fB}$	h	b	$A_{0}$
No.	Mpa	Mpa	Mpa	%	Mpa	%	Mpa	%	mm	mm	${mm}^{2}$
1	2750.00	55.4	66.5	2.7	61.4	2.9	52.0	3.1	6.05	13.38	80.95
2	2780.00	57.2	68.9	3.0	63.8	3.2	39.4	3.9	6.08	13.39	81.41
3	2720.00	53.4	69.4	3.1	60.3	3.0	51.0	3.3	6.08	13.41	81.53
4	2780.00	56.7	69.6	3.0	62.8	3.0	55.9	3.4	6.08	13.41	81.53
5	2630.00	55.4	63.6	3.2	61.4	3.1	51.5	3.4	6.08	13.40	81.47

Figure 11 shows graphs referring to the bending test results of the composition with the 5% filler of PB-GO represented in Table 10.

In the bending test, it was observed that the 3% and 5% filler compositions exhibited different trends in the average support stress and deformation. This could potentially be due to the dispersion of the additive within the matrix being less effective, particularly at lower concentrations, such as 3%.

At this level, it is possible that the additive forms agglomerates, which may act as stress concentration points, potentially weakening the matrix and reducing its ability to withstand mechanical stress. This suggests that the additive may not be uniformly distributed throughout the material, potentially leading to localized variations in its properties.

The ceramic nature of PB, in combination with the incorporation of GO, could potentially disturb the effective dispersion of the additive. This hypothesis is supported by X-ray analysis, which suggests that the additive’s dispersion is only partially effective, possibly due to incompatibilities in the selected sample or inherent limitations in the material’s characteristics.

**Figure 12:**
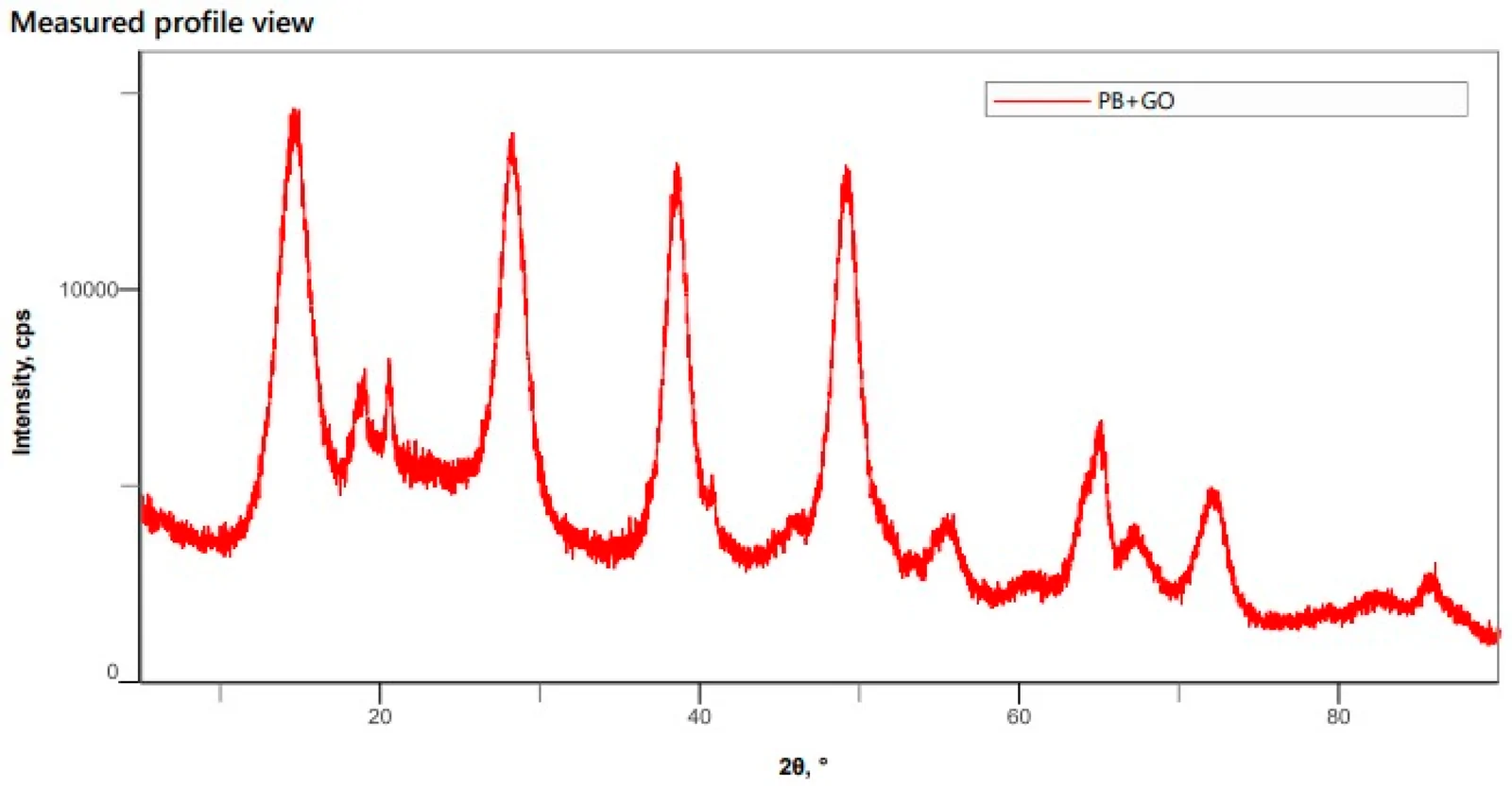
Result of the XRD test, indicating the Miller indices of the crystalline planes.

The X-ray diffraction test demonstrated that the peaks found for the charge match those found in the literature for pseudoboehmite. This result indicates that the process of coating graphene oxide with pseudoboehmite was successful. The result is shown in Figure 12.

It is possible that at higher concentrations, such as 5%, the dispersion of the additive improves, reducing the significance of agglomerates relative to the total volume of the material. This behavior may confirm that the primary issue lies in the state of dispersion rather than the bonding strength between the additive and the matrix.

Hardness tests revealed that the addition of the filler had little impact on the hardness of the material. The 3% filler composition showed a slight increase in the average hardness value by approximately 1%, while the 5% filler composition showed a slight increase in the average hardness, increasing by 1% compared to the 3% filler composition and increasing by 2% in relation to the composition of pure PLA. Test results are shown in Table 11, Table 12 and Table 13.

Despite these minor variations in hardness, when fillers are added, their main effects, such as changing the internal microstructure or improving interfacial bonding with the polymer, tend to influence tensile and bending strength mechanical properties more significantly, as shown in the tensile and flexural tests.

Hardness is primarily a surface property, reflecting only the localized resistance of the material to indentation or scratching. In contrast, properties such as tensile and bending strength depend on the entire volume of the material and involve stress distribution throughout the matrix.

As a result, even if the hardness values do not change substantially, modifications to factors like crystallinity, filler–matrix interaction, or load transfer mechanisms led to more pronounced alterations in tensile and bending performance.

HDT and VICAT analyses showed that the addition of the filler generated a slight drop in thermal deflection temperature from 51.8 °C to 50.2 °C in the 3% composition and to 51.4 °C in the 5% composition. There was also an increase in the VICAT temperature from 58.9 °C to 63.2 °C in the 3% composition and to 61.5 °C in the 5% composition. The increase in VICAT temperature may indicate that the load generated an increase in the Tg of the material. However, the drop in HDT indicates that the material became slightly less resistant with an increase in temperature. The HDT/VICAT results are shown in Table 14.

Inadequate dispersion, which can lead to particle agglomeration, may create local stress concentrations that facilitate deflection under load during heating, thereby slightly reducing the HDT. Although the nanofillers can locally restrict the molecular mobility of the polymer matrix, their low concentration (around 3%) limits the overall impact on the bulk material.

Nevertheless, the increased VICAT temperature observed in these systems suggests improved local thermal stability, as the presence of nanofillers reduces polymer chain mobility by effectively “locking” adjacent polymer sequences. This mechanism diminishes deformation when the material is exposed to heat because each interfacial bond acts as a barrier to heat flow, enhancing the composite’s resistance to thermal distortion.

## 4. Conclusions

In the literature, there are already works with PLA materials with PB fillers added and PLA with GO nanofillers added [27], but there are no PLA materials with both PB + GO fillers added simultaneously.

The GO nanofillers were well dispersed and bound to the PB matrix according to EDS and XRD analyses.

Comparing Figure 8, Figure 9 and Figure 10, there is an increase in yield stress, an increase in resilience, and an increase in toughness and ductility, which are good properties when PLA is used as a skin graft.

Still, in terms of skin scaffolds, flexion tests indicate an increase of between 5% and 10%, with real gains in tenacity, which is important for adaptation as grafts.

The presence of fillers practically does not reduce thermal deflection, as observed in HDT tests, and the body added with 3% PB + GO nanofillers presented the highest VICAT temperature, with an increase of approximately 8%.

As for the impact resistance test, there were no indications of an increase or decrease in relation to pure PLA, which is why it was not mentioned in the results.

In comparison, the PB + GO nanofiller in the 3% composition proved to be slightly more effective in terms of all its properties. Even though the PB+GO nanofiller with 5% in the composition showed better toughness, the PB+GO nanofiller with 3% in the composition made the scaffold more thermally stable.

In future works, in vitro and in vivo tests will be conducted to evaluate the effectiveness of the proposed PLA-based scaffold with the PB and GO presented in this article.

## Figures and Tables

**Table 1:** Zeta potential parameters.

Index	Mean Zeta Potencial (mV)	Conductivity (mS/cm)	Electrophoretic Mobility (µm.cm/Vs)	Adjusted Voltage (V)	Processed Runs	Color
1	27.3	0.064	2.1259	200	100	
2	26.9	0.062	2.1003	200	100	
3	28.4	0.062	2.2119	200	100	

**Table 2:** Impact test results for pure PLA.

Sample Number	Thickness	Width	Energy	Energy f (Thickness)	Energy f (Area)	Type of Fracture	Impact Speed
	mm	mm	J	J/m	$\mathrm{KJ}/m^{2}$		m
1	3.11	9.25	0.10189	32.762	3.542	Complete	3.45
2	3.43	9.67	0.10491	30.586	3.163	Complete	3.45
3	3.23	8.76	0.07491	23.191	2.647	Complete	3.45
4	3.25	9.68	0.08187	25.190	2.602	Complete	3.45
5	3.18	9.67	0.08985	28.255	2.922	Complete	3.45
6	3.12	9.52	0.07789	24.964	2.622	Complete	3.45
7	3.13	8.75	0.07292	23.298	2.663	Complete	3.45
6	3.21	8.82	0.07094	22.100	2.506	Complete	3.45
9	3.14	9.96	0.06896	21.962	2.205	Complete	3.45
10	3.13	9.69	0.10895	34.807	3.592	Complete	3.45
11	3.13	9.82	0.07292	23.298	2.373	Complete	3.45
12	3.14	8.87	0.10895	34.696	3.912	Complete	3.45

**Table 3:** Impact test results for the 3% composition.

Sample Number	Thickness	Width	Energy	Energy f (Thickness)	Energy f (Area)	Type of Fracture	Impact Speed
	mm	mm	J	J/m	$\mathrm{KJ}/m^{2}$		m
1	3.08	9.95	0.08286	26.903	2.704	Complete	3.45
2	3.12	9.86	0.08486	27.198	2.758	Complete	3.45
3	3.09	9.69	0.08585	27.785	2.867	Complete	3.45
4	3.08	9.85	0.08187	26.580	2.699	Complete	3.45
5	3.09	9.80	0.08985	29.078	2.967	Complete	3.45
6	3.09	9.77	0.08486	27.462	2.811	Complete	3.45
7	3.08	9.90	0.08685	28.199	2.848	Complete	3.45
8	3.07	9.82	0.07888	25.694	2.617	Complete	3.45
9	3.09	9.85	0.04048	13.100	1.330	Hinge	3.45
10	3.09	9.79	0.08087	26.172	2.673	Complete	3.45
11	3.08	9.88	0.08087	26.257	2.658	Complete	3.45
12	3.07	9.76	0.08187	26.667	2.732	Complete	3.45

**Table 4:** Impact test results for the 5% composition.

Sample Number	Thickness	Width	Energy	Energy f (Thickness)	Energy f (Area)	Type of Fracture	Impact Speed
	mm	mm	J	J/m	$\mathrm{KJ}/m^{2}$		m
1	3.08	9.95	0.08286	26.903	2.704	Complete	3.45
2	3.12	9.86	0.08486	27.198	2.758	Complete	3.45
3	3.09	9.69	0.08585	27.785	2.867	Complete	3.45
4	3.08	9.85	0.08187	26.580	2.699	Complete	3.45
5	3.09	9.80	0.08985	29.078	2.967	Complete	3.45
6	3.09	9.77	0.08486	27.462	2.811	Complete	3.45
7	3.08	9.90	0.08685	28.199	2.848	Complete	3.45
8	3.07	9.82	0.07888	25.694	2.617	Complete	3.45
9	3.09	9.85	0.04048	13.100	1.330	Hinge	3.45
10	3.09	9.79	0.08087	26.172	2.673	Complete	3.45
11	3.08	9.88	0.08087	26.257	2.658	Complete	3.45
12	3.07	9.76	0.08187	26.667	2.732	Complete	3.45

**Table 5:** Tensile test results for pure PLA.

	$A_{0}$	$E_{t}$	$E_{Sec}$	$\sigma_{x1}$	$\sigma_{y}$	$\sigma_{M}$	$\varepsilon_{M}$	$\varepsilon_{M(Corr.)}$	$\sigma_{B}$	$\varepsilon_{B}$	$\varepsilon_{B(Corr)}$	h	b
No.	${mm}^{2}$	MPa	MPa	MPa	Mpa	MPa	%	%	MPa	%	%	mm	mm
1	42.46	2580	2590.0	42.0	47.4	43.9	1.90	1.90	43.9	1.90	1.90	3.2	13.27
2	42.17	2560	2340.0	23.7	35.4	33.5	1.50	1.40	33.5	1.50	1.40	3.18	13.26
3	42.17	2400	2470.0	-	33.4	29.1	1.20	1.20	29.1	1.20	1.20	3.18	13.26
4	42.33	2310	2430.0	35.4	36.4	35.4	1.60	1.70	27.5	2.30	2.30	3.19	13.27
5	42.10	2430	2520.0	-	36.4	35.5	1.50	1.50	35.5	1.50	1.50	3.17	13.28
6	43.19	2890	2567.0	26.0	27.6	26.9	0.99	1.00	26.9	0.99	1.00	3.25	13.29
7	42.03	2450	2490.0	-	37.0	36.1	1.50	1.50	36.1	1.50	1.50	3.17	13.26
8	43.13	2350	2520.0	-	22.4	21.6	0.86	0.90	21.6	0.86	0.90	3.25	13.27
9	43.46	2560	2522.0	-	17.5	14.2	0.56	0.56	14.2	0.56	0.56	3.27	13.29
10	43.99	2740	2566.6	27.0	28.1	25.4	0.91	0.92	25.4	0.91	0.92	3.31	13.29
11	42.60	2680	2570.0	28.4	28.0	27.6	1.10	1.10	27.6	1.10	1.10	3.21	13.27
12	42.17	2700	2580.0	29.6	35.0	33.5	1.40	1.40	33.5	1.40	1.40	3.18	13.26

**Table 6:** Tensile test results for compositions with the 3% filler of PB-GO.

	$A_{0}$	$E_{t}$	$E_{Sec}$	$\sigma_{x1}$	$\sigma_{y}$	$\sigma_{M}$	$\varepsilon_{M}$	$\varepsilon_{M(Corr.)}$	$\sigma_{B}$	$\varepsilon_{B}$	$\varepsilon_{B(Corr)}$	h	b
No.	${mm}^{2}$	MPa	MPa	MPa	MPa	MPa	%	%	MPa	%	%	mm	mm
1	41.71	2390	2390.0	31.8	36.4	35.9	1.80	1.90	35.9	1.80	1.90	3.15	13.24
2	41.61	2290	2250.0	30.5	-	34.7	1.80	1.80	34.7	1.80	1.80	3.14	13.25
3	41.87	2130	2280.0	37.3	-	37.6	1.90	1.90	37.6	1.90	1.90	3.16	13.25
4	41.97	2600	2520.0	29.5	39.5	36.8	1.80	1.80	36.8	1.80	1.80	3.17	13.24
5	41.87	2830	2550.0	25.6	35.1	36.1	1.70	1.70	36.1	1.70	1.70	3.16	13.25
6	41.87	2350	2390.0	33.6	39.5	36.4	1.70	1.80	36.4	1.70	1.80	3.18	13.25
7	41.87	2540	2420.0	28.2	39.7	35.6	1.80	1.80	35.6	1.80	1.80	3.16	13.25
8	42.17	3390	2400.0	26.0	32.1	12.1	0.44	0.43	12.1	0.44	0.43	3.18	13.26
9	42.14	2570	2330.0	23.4	33.0	34.7	1.70	1.70	34.7	1.70	1.70	3.18	13.25
10	42.60	2470	2390.0	30.9	36.0	34.9	1.70	1.70	34.9	1.70	1.70	3.22	13.23

**Table 7:** Tensile test results for compositions with the 5% filler of PB-GO.

	$A_{0}$	$E_{t}$	$E_{Sec}$	$\sigma_{x1}$	$\sigma_{y}$	$\sigma_{M}$	$\varepsilon_{M}$	$\varepsilon_{M(Corr.)}$	$\sigma_{B}$	$\varepsilon_{B}$	$\varepsilon_{B(Corr)}$	h	b
No.	${mm}^{2}$	Mpa	Mpa	Mpa	Mpa	Mpa	%	%	Mpa	%	%	mm	mm
1	42.17	3140	2640.0	20.1	42.5	36.7	1.70	1.70	36.7	1.70	1.70	3.18	13.26
2	41.71	2880	2520.0	21.7	42.3	39.1	1.90	1.90	39.1	1.90	1.90	3.15	13.24
3	41.74	2570	2480.0	29.2	42.0	38.3	1.90	1.90	38.3	1.90	1.90	3.15	13.25
4	41.57	2930	2570.0	24.4	41.3	41.3	2.00	2.00	37.5	2.50	2.50	3.14	13.24
5	42.00	2730	2570.0	29.1	40.0	37.8	1.90	1.80	37.8	1.90	1.80	3.17	13.25
6	41.87	2840	2600.0	26.9	39.5	39.5	1.90	1.90	39.5	1.90	1.90	3.16	13.25
7	41.71	2970	2640.0	24.4	40.0	38.9	1.90	1.90	38.9	1.90	1.90	3.15	13.24
8	41.54	2790	2570.0	27.4	42.1	39.9	1.90	1.90	39.9	1.90	1.90	3.14	13.23
9	41.54	3000	2620.0	23.0	41.9	40.8	2.00	2.00	40.8	2.00	2.00	3.14	13.23
10	41.64	2790	2580.0	26.5	39.6	38.9	2.00	2.00	38.9	2.00	2.00	3.15	13.22

**Table 8:** Bending test results for pure PLA.

	$E_{r}$	$\sigma_{0.2}$	$\sigma_{fY}$	$\varepsilon_{fY}$	$\sigma_{fM}$	$\varepsilon_{fM}$	$\sigma_{fB}$	$\varepsilon_{fB}$	h	b	$A_{0}$
No.	MPa	MPa	MPa	%	MPa	%	MPa	%	mm	mm	${mm}^{2}$
1	2530.00	21.5	-	1.0	25.9	1.0	21.4	1.1	6.05	13.30	80.47
2	2790.00	57.8	58.9	2.5	58.9	2.5	11.8	2.0	6.09	13.37	81.42
3	2740.00	55.5	56.8	2.6	56.8	2.6	11.4	1.5	6.06	13.38	81.08
4	2610.00	55.5	57.3	2.6	57.3	2.6	11.0	2.4	6.09	13.35	81.30
5	2780.00	54.1	55.0	1.9	54.1	2.2	10.8	2.6	6.08	13.40	81.47

**Table 11:** Hardness test results for pure PLA.

PLA Pure		PLA Pure		PLA Pure	
Test Body Lote 1		Test Body Lote 2		Test Body Lote 3	
Ponto	Hardn	Ponto	Hardn	Ponto	Hardn
1	79.0	1	80.0	1	81.0
2	80.0	2	79.0	2	80.0
3	79.0	3	80.0	3	80.0
4	79.0	4	81.0	4	80.0
5	79.0	5	80.0	5	80.0
Av. Hardn.	79.4	Av. Hardn.	80.0	Av. Hardn.	80.2

**Table 12:** Hardness test results for the 3% filler composition.

PLA with 3% Filler		PLA with 3% Filler		PLA with 3% Filler	
Test Body Lote 1		Test Body Lote 2		Test Body Lote 3	
Ponto	Hardn	Ponto	Hardn	Ponto	Hardn
1	81.0	1	81.0	1	82.0
2	82.0	2	82.0	2	81.0
3	81.0	3	82.0	3	81.0
4	81.0	4	81.0	4	81.0
5	81.0	5	81.0	5	81.0
Av. Hardn.	81.2	Av. Hardn.	81.4	Av. Hardn.	81.2

**Table 13:** Hardness test results for the 5% filler composition.

PLA with 5% Filler		PLA with 5% Filler		PLA with 5% Filler	
Test Body Lote 1		Test Body Lote 2		Test Body Lote 3	
Ponto	Hardn	Ponto	Hardn	Ponto	Hardn
1	83.0	1	82.0	1	83.0
2	84.0	2	83.0	2	83.0
3	84.0	3	81.0	3	83.0
4	83.0	4	81.0	4	82.0
5	83.0	5	82.0	5	82.0
Av. Hardn.	83.4	Av. Hardn.	81.8	Av. Hardn.	82.6

**Table 14:** HDT/VICAT test results.

	PLA Pure		PLA + 3% (PB+GO)			PLA + 5% (PB+GO)		
Test Body	HDT (°C)	VICAT (°C)	Test Body	HDT (°C)	VICAT (°C)	Test Body	HDT (°C)	VICAT (°C)
1	51.8	58.1	1	49.7	63	1	51.7	61.4
2	52.1	59	2	51.1	63.5	2	51.2	61.6
3	51.4	58.9	3	48.8	63	3	51.3	61.3
4	51.9	58.8	4	51.3	63.3	4	51.5	61.5
5	51.9	59.8	5	50.2	63	5	51.5	61.6
average	51.8	58.9	average	50.2	63.2	average	51.4	61.5

## Data Availability

Data is contained within the article.
