# Correction: Maidana et al. Synthesis and Characterization of a Pla Scaffold with Pseudoboehmite and Graphene Oxide Nanofillers Added. *Nanomaterials* 2025, *15*, 167

**DOI:** 10.3390/nano16181167

**Published:** 2026-09-16

**Authors:** Rafael Vieira Maidana, Antônio Hortêncio Munhoz, Filipe Figueiredo Ramos, Alex Lopes de Oliveira, José César de Souza Almeida Neto, Victor Inácio de Oliveira, Bruno Luis Soares de Lima, Fábio Jesus Moreira de Almeida

**Affiliations:** Engineering School, Mackenzie Presbyterian University, 930 Consolação Street, Consolação, São Paulo 01302-907, SP, Brazil; rafael.maidana@mackenzista.com.br (R.V.M.); antonio.munhoz@mackenzie.br (A.H.M.J.); filipe.ramos@mackenzie.br (F.F.R.); alex.oliveira@mackenzie.br (A.L.d.O.); jose.almeida@mackenzie.br (J.C.d.S.A.N.); victor.inacio@mackenzie.br (V.I.d.O.); bruno.lima@mackenzie.br (B.L.S.d.L.)

In the original publication [1], there was an error concerning the interpretation of rubidium (Rb) peak and technetium (Tc) signals. The authors wish to update the text and add definitions of the abbreviations at their first occurrence in Section 3, paragraph 3 as follows:

“The Energy Dispersive Spectroscopy (EDS) analysis of graphene oxide (GO) showed that the analyzed material was predominantly composed of carbon and oxygen. The EDS software also generated small automatic indications assigned to rubidium (Rb) and technetium (Tc). However, these assignments were not independently confirmed. Both indications occurred in spectrally crowded regions susceptible to peak overlap, background effects, low-count statistical noise, and automatic misidentification. The non-conductive sample was coated with gold before SEM/EDS analysis, and the Au M-lines provide a known spectral contribution in the same low-energy region. In addition, technetium is not chemically expected in commercial GO, PLA, pseudoboehmite, or the reagents and preparation materials used in this study, and no plausible technetium source existed in the experimental process. The corresponding software-generated ‘Tc L’ map was weak, speckled, and showed no coherent spatial correlation with the sample morphology. Accordingly, neither the Rb nor the Tc indication should be interpreted as evidence that these elements were genuine constituents of the sample. They are therefore treated as unconfirmed automatic software assignments/artifacts, and the material is interpreted as being predominantly composed of carbon and oxygen, as presented in Figure 3.”

Corrections have been made in the Author Contributions section in the back matter as follows:

**Author Contributions:** Conceptualization, F.J.M.d.A. and A.H.M.J.; Methodology, A.H.M.J., A.L.d.O. and R.V.M.; Investigation, R.V.M., F.F.R., J.C.d.S.A.N. and V.I.d.O.; Validation, A.H.M.J., J.C.d.S.A.N. and V.I.d.O.; Formal Analysis, F.J.M.d.A., A.L.d.O. and R.V.M.; Data Curation, F.F.R. and V.I.d.O.; Resources, A.H.M.J. and B.L.S.d.L.; Project Administration, B.L.S.d.L.; Visualization, F.F.R. and J.C.d.S.A.N.; Writing—Original Draft Preparation, F.J.M.d.A.; Writing—Review and Editing, F.J.M.d.A., A.L.d.O., F.F.R., A.H.M.J. and B.L.S.d.L.; Supervision, F.J.M.d.A. and A.H.M.J. All authors have read and agreed to the published version of the manuscript and to the proposed correction.

The authors state that the scientific conclusions are unaffected. This correction was approved by the Academic Editor. The original publication has also been updated.
